# Decision‐theoretic designs for a series of trials with correlated treatment effects using the Sarmanov multivariate beta‐binomial distribution

**DOI:** 10.1002/bimj.201600202

**Published:** 2017-07-26

**Authors:** Siew Wan Hee, Nicholas Parsons, Nigel Stallard

**Affiliations:** ^1^ Statistics and Epidemiology Division of Health Sciences Warwick Medical School University of Warwick Coventry CV4 7AL UK

**Keywords:** backward induction, Bayesian decision theory, bivariate beta distribution, correlated trials, Sarmanov beta‐binomial

## Abstract

The motivation for the work in this article is the setting in which a number of treatments are available for evaluation in phase II clinical trials and where it may be infeasible to try them concurrently because the intended population is small. This paper introduces an extension of previous work on decision‐theoretic designs for a series of phase II trials. The program encompasses a series of sequential phase II trials with interim decision making and a single two‐arm phase III trial. The design is based on a hybrid approach where the final analysis of the phase III data is based on a classical frequentist hypothesis test, whereas the trials are designed using a Bayesian decision‐theoretic approach in which the unknown treatment effect is assumed to follow a known prior distribution. In addition, as treatments are intended for the same population it is not unrealistic to consider treatment effects to be correlated. Thus, the prior distribution will reflect this. Data from a randomized trial of severe arthritis of the hip are used to test the application of the design. We show that the design on average requires fewer patients in phase II than when the correlation is ignored. Correspondingly, the time required to recommend an efficacious treatment for phase III is quicker.

## 1 Background

Before it is authorized by regulatory authorities, a new treatment is first tested for tolerability in phase I followed by an exploration of therapeutic effects in phase II and finally, efficacy and safety are assessed in phase III (ICH, 1997). The objective for each phase is different and traditionally each study is designed separately. For example, a phase I drug trial might be designed to identify the maximally tolerated dose and use a group of participants (usually healthy volunteers) at each dose level of a new drug whereas a nondrug trial may focus on safety assessments only. Once tolerability and safety are established, an exploratory phase II trial is undertaken to test efficacy against a standard treatment. This may be a single‐arm study where observed efficacy for the test treatment is compared to a fixed and known control, usually a standard treatment. After the test treatment has been shown to be reasonably efficacious it is compared to a control (standard treatment or placebo) in a phase III trial. The phase III trial is typically designed as a two‐arm parallel group randomized controlled trial where patients are randomized to either the test treatment or the control arm (Pocock, 1983).

There is often more than one new intervention (e.g., treatment or procedure) available for testing. This is problematic for the conventional paradigm of fixed and distinct phases composed of simple two‐arm comparisons. This difficulty is further compounded where resources such as patients and money are limited. This is especially true in clinical contexts with small research populations, for example, a rare disease or for a specifically targeted subpopulation. In such constrained settings, it is necessary to design trials in the context of other treatments or trials such that the management and allocation of limited resources may be optimized and research undertaken as efficiently as possible (Senn, 1996).

In this paper, we propose a design motivated by the scenario in which two or more treatments are available for clinical testing with the intended study population sufficiently small, or resources sufficiently limited, that it is not feasible to test all treatments concurrently. The proposed design is for a series of phase II run one after another and a single phase III trial using treatments that have been previously tested for tolerability and safety. A decision is made at the end of each phase II trial whether to accept the test treatment for further study (phase III) or to reject it. In making the latter decision, the decision maker may decide to start a new phase II trial with a different treatment or abandon the entire development plan. As it is essentially a decision problem whether or not to accept the test treatment for further study, statistical decision theory seems an obvious choice to model the design of the phase II trial (Julious & Swank, 2005). We propose such an approach that allows the quantification of the reward (or gain) from a future successful phase III trial, if the treatment is accepted for further study, and losses incurred in conducting the trials. Note that the problem we are investigating is an elaboration of the well‐known *Secretary Problem* where one could only appoint one candidate from the known *n* candidates to the secretarial position, see, Ferguson (1989a) and commentaries and responses therein (Ferguson, 1989b; Freeman, 1989; Robbins, 1989; Sakaguchi, 1989; Samuels, 1989) for a summary of the problem, solutions, and extensions.

There is a relatively limited literature on methods for designing a program of phase II and III trials (see, e.g., Chen & Beckman, 2009; Ding, Rosner, & Müller, 2008; Hee & Stallard, 2012; Pallay, 2001; Rossell, Müller, & Rosner, 2007; Stallard, 2003, 2012; Stallard & Thall, 2001; Wason, Jaki, & Stallard, 2013; Whitehead, 1986). Of these, eight designs are based on a decision‐theoretic approach, (see, Hee et al., 2015 for a summary of their methods) in which by considering the probable success of the future of a recommended treatment in a phase III setting, the sample size for each phase II trial can be optimized. A working group from the Drug Information Association (DIA) Adaptive Design Scientific Working Group has been working on similar designs for a program of phase II and III trials in specific disease areas, namely, diabetes, oncology, and neuropathic pain (see, Antonijevic et al., 2013; Marchenko et al., 2013; Patel et al., 2012).

The method proposed here extends that was proposed by Hee and Stallard (2012). They considered a program consisting of a series of sequential phase II trials where at each interim stage a decision is made to continue with recruitment to the current trial, stop and proceed to a phase III trial with the current treatment, stop and initiate a phase II trial with a new treatment or stop and abandon the entire program. Unknown parameters were assumed to follow a specified prior distribution and were assumed by Hee and Stallard to be independent. In reality, however, treatments targeting the same population may be related. In this case, information from earlier treatments may inform our opinion of subsequent treatments, which in turn affects their optimal decision schemes. Therefore, in this paper, we propose an extension which allows correlation between the efficacy parameters for the test treatments.

## 2 Framework

### 2.1 Setting

Following Hee and Stallard (2012), we assume at the design stage of a clinical research program that the size of the study population and the number of treatments available for testing are known and fixed. We plan to conduct a series of single‐arm phase II trials followed by a single randomized controlled phase III trial. We assume that the primary endpoints for both phase II and III trials are the same and that the responses follow a Bernoulli distribution.

The terms program and development plan are used interchangeably to represent this series of trials. The phase II trials are conducted one after another with interim analyses. We also assume that treatments already tested in the current program will not be evaluated further and patients previously recruited into the current program will not join future trials of the current program.

Within each phase II trial, a group of patients is treated in each interim stage and a decision is made based on their observed responses from the following actions:

Action A: Stop the current phase II trial and abandon the entire program;
Action P: Stop the current phase II trial and proceed to a two‐arm phase III trial with this test treatment compared to the standard treatment using all the remaining patients;
Action T: Stop the current phase II trial and initiate a new single‐arm phase II trial for a different treatment; or
Action R: Continue the current phase II trial by recruiting additional patients.

Not all four actions are available for consideration at all interim stages; if all available treatments have been tried then action T is not available at any interim stage of the trial of the final treatment. We also restrict the phase III trial to a minimum sample size. Thus when only this minimum number of patients remain from the original research population, actions R and T are not available.

It is assumed that at the end of the phase III trial, the observed data from the trial are analyzed based on a frequentist hypothesis test. However, in the design stage of the whole development plan we will adopt a Bayesian approach in which the unknown treatment efficacy parameters are assumed to be random and follow a known prior distribution. That is, the design is based on a hybrid approach, a combination of classical and Bayesian frameworks.

### 2.2 Model

Let *N* denote the total size of the population eligible for both phase II and III trials and *K* denote the number of test treatments with *N* and *K* assumed fixed and known. Let *n*_III, min_ be the predetermined minimum number of patients required for the phase III trial and $m_{ki}$ be the predetermined fixed number of patients recruited at the *i*‐th stage of the *k*‐th phase II trial, k=1,…,K. The total number of patients recruited from the first stage up to and including the *i*‐th stage of the *k*‐th trial is $n_{ki}=\sum_{j=1}^{i}m_{kj}$. Assuming patients' responses to be independent Bernoulli random variables, let $Y_{ki}$ be the number of successes out of the $m_{ki}$ patients in *i*‐th stage of trial *k* and $S_{ki}=\sum_{j=1}^{i}Y_{kj}$ be the accumulated total number of successes out of $n_{ki}$ patients from the first *i* stages in trial *k* so that $S_{ki}∼Bin\left(n_{ki},p_{k}\right)$ for $p_{k}$, and similarly, $Y_{ki}∼Bin\left(m_{ki},p_{k}\right)$. Given $p_{k}$, the density function of $S_{ki}$ and $Y_{ki}$ are fS|p(ski|nki,pk)=nkiskipkski(1−pk)nki−ski and $f(y_{ki}|m_{ki},p_{k})$, respectively.

Suppose $n_{k+}$ is the total number of patients in trial *k* and $S_{k+}$ is the total number of successes from the $n_{k+}$ patients when action T (stop the current phase II trial and start another with a new test treatment) is taken. During the conduct of the current *k*‐th trial, there is no observation from future trials. Let $n_{\mathrm{ki}}=\left(n_{1+},\ldots ,n_{k-1,+},n_{ki},0,\ldots ,0\right)$ be the random vector of the number of patients recruited from the preceding trials and from the first stage up to and including the *i*‐th stage of the *k*‐th trial and $S_{\mathrm{ki}}=\left(S_{1+},\ldots ,S_{k-1,+},S_{ki},0,\ldots ,0\right)$ be the corresponding vector of successes observed. Let$S_{k0}=\left(S_{1+},\ldots ,S_{k-1,+},0,\ldots ,0\right)$ denote the vector of successes out of $n_{k0}=\left(n_{1+},\ldots ,n_{k-1,+},0,\ldots ,0\right)$ patients observed from the preceding trials at the start of the *k*‐th trial; this will be referred to as stage 0. The joint conditional distribution of $S_{\mathrm{ki}}=s_{\mathrm{ki}}$ given $n_{\mathrm{ki}}$ and $p=(p_{1},\ldots ,p_{K})$ at stage *i* in trial *k* is the product of density functions, $h_{S_{\mathrm{ki}}|p}\left(S_{\mathrm{ki}}=s_{\mathrm{ki}}|p\right)=\prod_{k^{\prime }=1}^{k-1}f_{S|p}\left(s_{k^{\prime }+}|n_{k^{\prime }+},p_{k^{\prime }}\right)f_{S|p}\left(s_{ki}|n_{ki},p_{k}\right)$. Note that at stage $i=0,n_{ki}=0$ and we take $f_{S|p}\left(0|0,p_{k}\right)=1$.

In this proposed design, we assume that the parameters p follow some joint prior distribution denoted by the *K*‐variate joint density, $h_{p}\left(p\right)$. Upon observing the accumulated successes, $s_{\mathrm{ki}}$, information on p is updated and the joint posterior density obtained using Bayes' theorem is $h_{p|S_{\mathrm{ki}}}\left(p|s_{\mathrm{ki}}\right)=h_{S_{\mathrm{ki}}|p}\left(s_{\mathrm{ki}}|p\right)h_{p}\left(p\right)/h_{S_{\mathrm{ki}}}\left(s_{\mathrm{ki}}\right)$ where $h_{S_{\mathrm{ki}}}\left(s_{\mathrm{ki}}\right)=\int \;\cdots \;\int h_{S_{\mathrm{ki}}|p}\left(s_{\mathrm{ki}}|p\right)h_{p}\left(p\right)\;dp$, is the marginal joint density of $S_{\mathrm{ki}}$.

We have noted earlier that patients' responses are conditionally independent given parameters p. Therefore, suppose we have observed $S_{\mathrm{ki}}=s_{\mathrm{ki}}$, the posterior marginal density of $Y_{k,i+1}$ is

(1) $$g_{Y|S_{\mathrm{ki}}}(y_{k,i+1}|s_{\mathrm{ki}},n_{\mathrm{ki}})=\int \;\cdots \;\int f(y_{k,i+1}\left|m_{k,i+1},p_{k})h_{p|S_{\mathrm{ki}}}(p\right|s_{\mathrm{ki}})\;dp.$$

## 3 Utilities and backward induction

### 3.1 Utilities

At each stage of each trial, the desirability of each action is expressed by some gain or utility function capturing rewards and costs which will be assumed to depend on the true parameters, p, the total number of patients, *N*, and the numbers of patients recruited so far, $n_{\mathrm{ki}}$. The reward may be the monetary value of taking the action or it could be a value that has no obvious scale of measurement, such as the value of treatment effectiveness. The cost of conducting a trial is separated into fixed and variable costs (Patel & Ankolekar, 2007). The costs of phase II and III trials may be different due, in part at least, to longer follow‐up and more comprehensive outcome assessment in studies of the latter type. The fixed costs are costs that are not dependent on the size of the trial. Let l II ,k and l III ,k denote the costs of setting up and conducting phase II and III trials for treatment *k*, respectively. The variable cost, on the other hand, depends on the size of the trial and can be expressed as the cost per patient. Let c II ,k and c III ,k denote the cost per patient in a phase II and III trials, respectively, for treatment *k*.

Let $G_{a}\left(k,p,n_{\mathrm{ki}},N\right)$ be the gain function for action a,a∈{A,P,T,R}, in trial *k* given the sample sizes $n_{\mathrm{ki}}$ and the true value of p. The gain function for each action is described in detail in Sections 4.1 to 4.3. As the true parameters are unknown, the expected utility function of action *a* can be obtained as the expectation over p. Having observed responses from the preceding trials and from the first stage up to and including the *i*‐th stage of trial *k*, the expected utility of action *a* is the expectation over the joint posterior density of p given $s_{\mathrm{ki}}$. For a∈{A,P}, the expected utility is given by $G_{a}\left(k,s_{\mathrm{ki}},n_{\mathrm{ki}},N\right)=E\left(G_{a}\left(k,p,n_{\mathrm{ki}},N\right)|s_{\mathrm{ki}}\right)=\int \;\cdots \;\int G_{a}\left(k,p,n_{\mathrm{ki}},N\right)h_{p|S_{\mathrm{ki}}}\left(p|s_{\mathrm{ki}}\right)\;dp$. The expected utility function for a∈{T,R} depends on the expected utility of subsequent actions and we show how they are calculated below (see Sections 4.2 and 4.3). The optimal action at stage *i* of trial *k* is the one that maximizes the expected gain function, ${\arg\max}_{a\in \{A,P,T,R\}}G_{a}\left(k,s_{\mathrm{ki}},n_{\mathrm{ki}},N\right)$.

## 4 Expected utility functions

The utility function is defined relative to an arbitrary reference value (Hilden, 1990). A natural and convenient one is to fix it to the initial stage of the current trial where no cost is spent. This is because the utility function within each trial does not need to account for the costs incurred from previous treatments as these costs are common constants during the comparisons of actions at each stage of the current treatment.

### 4.1 Expected utility of action A (abandon the program) and action P (proceed to a phase III trial)

Following the design by Hee and Stallard (2012) the utility function we consider in this work is motivated from a commercial perspective and the expected utility functions of actions A and P have similar forms to those described by them;

(2) Ga(k,ski,nki,N)=−c II ,knki,a=A,∫⋯∫U1−Φ(z1−α/2−θkVk)hp|Ski(p|ski)dp+−c II ,knki−c III ,kN−∑j=1k−1nj+−nki−l III ,k,a=P,

where *U* is the gain of identifying the experimental treatment as more effective than the control treatment correctly, $\theta_{k}=\log\{p_{k}\left(1-p_{C}\right)/\left(p_{C}\left(1-p_{k}\right)\right)$ is the log odds ratio, Φ(·) is the cumulative standard normal distribution function, $z_{\gamma }$ is the upper 100γ percentile of the standard normal density, and *V* is Fisher's information. The gain *U* can be a fixed constant value or a function of θ where the gain depends on the level of efficacy of the experimental treatment (see, e.g., Berry & Ho, 1988). The notable difference in our proposed design from Hee and Stallard (2012) is that the expected utility of action P is the expectation over the joint posterior density.

### 4.2 Expected utility of action T (start a new phase II trial)

If action T is taken after observation of $n_{\mathrm{ki}}$ patients, the *k*‐th trial is abandoned and a new phase II trial is initiated with the remaining population of size $N-\sum_{j=1}^{k-1}n_{j+}-n_{ki}$. The expected utility of action T depends on the expected utility of the new trial and its resulting actions may be found by backward induction (as follows). The expected utility of action T is the expected utility of the (k+1)‐th trial less the cost of patients recruited thus far to the *k*‐th trial, that is

(3) GT(k,ski,nki,N)=G Total (k+1,sk+1,0,nk+1,0,N)−c II ,knki,

where G Total (k+1,sk+1,0,nk+1,0,N) is the expected utility of the (k+1)‐th trial having observed $S_{k+1,0}=\left(S_{1+},\ldots ,S_{k+},0,\ldots ,0\right)$ successes out of $n_{k+1,0}=\left(n_{1+},\ldots ,n_{k+},0,\ldots ,0\right)$ patients from the first *k* trials and is calculated as shown below (Section 4.4).

We set $G_{T}\left(k,s_{\mathrm{ki}},n_{\mathrm{ki}},N\right)=-\infty$ if ∑j=1k−1nj++nki≥N−n III , min  so that action T will not be chosen as the optimum action at stage *i* of trial *k* since this would leave fewer than the required minimum number of patients for a phase III trial at any point in the future. Similarly, when k=K, action T is not available at any interim stage and $G_{T}\left(k,s_{\mathrm{Ki}},n_{\mathrm{Ki}},N\right)=-\infty$ for all *i*.

### 4.3 Expected utility of action R (continue the current phase II trial)

Action R is to continue the current single‐arm phase II trial. The gain from action R after observation of $n_{\mathrm{ki}}$ patients from the preceding trials and from the first stage up to and including the *i*‐th stage of trial *k* depends on the action taken based on the observation from patients recruited in subsequent stages and trials. Consequently, the expected utility of action R is also obtained by backward induction. Suppose we recruit an additional $m_{k,i+1}$ patients to the current trial with $Y_{k,i+1}=y_{k,i+1}$ observed at the (i+1)‐th stage. The optimal action at this stage is then the action with the highest expected utility function, $\max_{a\in \{A,P,T,R\}}\left\{G_{a}\left(k,s_{\mathrm{ki}}+y_{k,i+1},n_{\mathrm{ki}}+m_{k,i+1},N\right)\right\}$. The expected utility of action R is the gain from recruiting the additional $m_{k,i+1}$ patients averaged over the possible responses given the observed successes from the preceding trials and from the first stage up to and including the *i*‐th stage of the current trial, that is

(4) $$\begin{array}{ccc} G_{R}(k,s_{\mathrm{ki}},n_{\mathrm{ki}},N) & = & \sum_{y_{k,i+1}=0}^{m_{k,i+1}}\max_{a\in \{A,P,T,R\}}\{G_{a}(k,s_{\mathrm{ki}}+y_{k,i+1},n_{\mathrm{ki}}+m_{k,i+1},N)\}\times \\ & & \times \;g_{Y|S_{\mathrm{ki}}}(y_{k,i+1}|s_{\mathrm{ki}},n_{\mathrm{ki}}), \end{array}$$

where $g_{Y|S_{\mathrm{ki}}}\left(y_{k,i+1}|s_{\mathrm{ki}},n_{\mathrm{ki}}\right)$ is the posterior marginal density of $Y_{k,i+1}$ given $S_{\mathrm{ki}}=s_{\mathrm{ki}}$ as given in (1). We set $G_{R}\left(k,s_{\mathrm{ki}},n_{\mathrm{ki}},N\right)=-\infty$ if ∑j=1k−1nj+nki≥N−n III , min ,k=1,…,K so that action R is not possible.

### 4.4 Expected utility of the *k*‐th trial

The *k*‐th trial starts by recruiting $m_{k1}$ patients to the first stage. The expected utility of the *k*‐th trial is obtained by considering the desirability of sampling $m_{k1}$ patients and the possible resulting actions, namely, actions A, P, T and R. The expected utilities of these actions are given, respectively, by Eqs. (2)–(4) which in turn depend on the expected utilities of subsequent actions. As there is a finite sequence of interim stages in the trial the expected utility of the *k*‐th trial is solved by computing the expected utilities of all actions at the ultimate stage for all possible responses. At this last stage, the expected utilities of actions A and P are given by (2), whereas expected utilities of actions T and R are set to −∞ as discussed in Sections 4.2 and 4.3. The expected utility functions for all actions at the penultimate stage are then solved based on the values from the ultimate stage and for all possible responses prior to the penultimate stage. Working methodically in this iterative manner the expected utility of the *k*‐th trial is obtained by backward induction. The computation of the expected utility of the trial and optimum sequential scheme is similar to the one described in Hee and Stallard (2012).

Suppose at the start of trial $k,S_{k0}=\left(S_{1+},\ldots ,S_{k-1,+},0,\ldots ,0\right)$ successes out of $n_{k0}=\left(n_{1+},\ldots ,n_{k-1,+},0,\ldots ,0\right)$ patients were observed from the preceding trials, the expected utility of the *k*‐th trial is determined by maximizing the expected utility of each possible action (i.e., averaging it over all possible values of $Y_{k1}$) less the cost of conducting the *k*‐th phase II trial. The expected utility is written as

G Total (k,sk0,nk0,N)=∑yk1=0mk1maxa∈{A,P,T,R}Ga(k,sk1,nk1,N)gY|Sk0(yk1|sk0,nk0)−l II ,k,

where $g_{Y|S_{k0}}\left(y_{k1}|s_{k0},n_{k0}\right)$ is the posterior marginal density of $Y_{k1}$ given $s_{k0}$, obtained as shown in (1), that is, $g_{Y|S_{k0}}\left(y_{k1}|s_{k0},n_{k0}\right)=\int \;\cdots \;\int f\left(y_{k1}|m_{k1},p_{k}\right)h_{p|S_{\mathrm{ki}}}\left(p|s_{k0}\right)\;dp$ and the individual expected utilities, $G_{a}\left(k,s_{k1},n_{k1},N\right)$, are given above (Sections 4.1–4.3). At the first stage of trial $k,S_{k1}=Y_{k1}$ and $n_{k1}=m_{k1}$, therefore, $S_{k1}=\left(S_{1+},\ldots ,S_{k-1,+},Y_{k1},0,\ldots ,0\right)$ and $n_{k1}=\left(n_{1+},\ldots ,n_{k-1,+},m_{k1},0,\ldots ,0\right)$.

The expected utility of the whole program G Total (s10,n10,N), is then obtained. If this is greater than 0 then it is worthwhile to start the trial by recruiting the first *m*_11_ patients to the first trial. Otherwise, the optimal decision is not to start the program at all.

## 5 Prior distribution

One possible form of the *K*‐variate beta distribution is one that follows the multivariate distribution family introduced by Sarmanov, described by Lee (1996). The joint density function is

(5) $$h_{p}\left(p_{1},\ldots ,p_{K}\right)=\left(\prod_{k=1}^{K}f_{p}\left(p_{k}\right)\right)\left(1+R_{\Omega_{K}}\left(p_{1},\ldots ,p_{K}\right)\right),$$

with

$$\begin{array}{cc} & R_{\Omega_{K}}\left(p_{1},\ldots ,p_{K}\right)=\sum_{j_{1}=1}^{K-1}\sum_{j_{2}=j_{1}+1}^{K}\omega_{j_{1},j_{2}}\phi (p_{j_{1}})\phi (p_{j_{2}})\;+\\ & \;+\sum_{j_{1}=1}^{K-2}\sum_{j_{2}=j_{1}+1}^{K-1}\sum_{j_{3}=j_{2}+1}^{K}\omega_{j_{1},j_{2},j_{3}}\phi (p_{j_{1}})\phi (p_{j_{2}})\phi (p_{j_{3}})+\cdots +\omega_{1,2,\ldots ,K}\prod_{j=1}^{K}\phi (p_{j}), \end{array}$$

where $\phi (p_{k})$ is a nonconstant mixing function bounded by $\int_{-\infty }^{\infty }\phi \left(p_{k}\right)f_{p}\left(p_{k}\right)\;dp_{k}=0$ and the mixing parameter $\Omega_{K}=\{\omega_{j_{1},j_{2}},\omega_{j_{1},j_{2},j_{3}},\ldots ,\omega_{1,2,\ldots ,K}\}$. Lee proposed $\phi \left(p_{k}\right)=p_{k}-\mu_{k}$ where $\mu_{k}$ is the expected value of $p_{k}$. The elements of $\Omega_{K}$ are chosen such that they satisfy the condition $1+R_{\Omega_{K}}(p_{1},\ldots ,p_{K})\geq 0$ for all p and when each element equals zero, then all *K* treatments are independent.

The joint posterior density is

(6) $$h_{p|S}\left(p_{1},\ldots ,p_{K}|s_{1},\ldots ,s_{K}\right)=\left\{\prod_{k=1}^{K}f_{p|S}\left(p_{k}|s_{k}\right)\right\}\left\{\frac{1+R_{\Omega_{K}}\left(p_{1},\ldots ,p_{K}\right)}{1+D_{\Omega_{K}}\left(s_{1},\ldots ,s_{K}\right)}\right\}$$

where $f_{p|S}\left(p_{k}|s_{k}\right)=p_{k}^{a_{k}+s_{k}-1}{\left(1-p_{k}\right)}^{b_{k}+n_{k}-s_{k}-1}/B\left(a_{k}+s_{k},b_{k}+n_{k}-s_{k}\right)$ is the marginal posterior density and

$$\begin{array}{cc} & D_{\Omega_{K}}(s_{1},\ldots ,s_{K})=\sum_{j_{1}=1}^{K-1}\sum_{j_{2}=j_{1}+1}^{K}\omega_{j_{1},j_{2}}\psi (s_{j_{1}})\psi (s_{j_{2}})\;+\\ & \;+\sum_{j_{1}=1}^{K-2}\sum_{j_{2}=j_{1}+1}^{K-1}\sum_{j_{3}=j_{2}+1}^{K}\omega_{j_{1},j_{2},j_{3}}\psi (s_{j_{1}})\psi (s_{j_{2}})\psi (s_{j_{3}})+\cdots +\omega_{1,2,\ldots ,K}\prod_{j=1}^{K}\psi (s_{j}), \end{array}$$

where $\psi (s_{k})=(s_{k}-\mu_{k}n_{k})/(a_{k}+b_{k}+n_{k})$.

## 6 Application

### 6.1 The Warwick arthroplasty trial (WAT)

In order to illustrate the characteristics of the development plan of a series of trials with correlated treatment efficacy we use data from the Warwick arthroplasty trial (WAT) (Costa et al., 2012). This was a two‐arm parallel group randomized controlled trial that assessed function after either total hip arthroplasty or resurfacing arthroplasty in patients with severe arthritis of the hip. The latter treatment group was the newer implant procedure (experimental) and the former the standard procedure (control). Patients were recruited between May 2007 and February 2010 and 60 of the total 126 patients were randomized to the experimental arm. One of the study endpoints was hip function at three months postsurgery assessed using the Oxford hip score (Dawson, Fitzpatrick, Carr, & Murray, 1996); this is often categorized into either poor (score, 0–26), fair (score, 27–33), good (score, 34–41), or excellent (score, 42–48) function (Costa et al., 2012).

Although there was only one experimental treatment in WAT, it is not uncommon in surgical trials to have more than one procedure that differ in the types of material and/or technical aspects of the operation. This design of a series of treatment where their effects are correlated is ideal for such surgical trials where the intended research population is small and there are more than one procedure available such that it is infeasible to try them concurrently.

### 6.2 Bivariate case

In our illustration, we assume that there are two (K=2) newer resurfacing arthroplasty procedures that differ in the technical aspects of the operation available for single‐arm phase II clinical trial and only one can proceed to a phase III trial where the control treatment would be total hip arthroplasty. We also assume that the primary endpoint is a binary outcome where a good or excellent function of the Oxford hip score at three months postsurgery is considered as a success (represented numerically as 1) whereas a poor or fair function is considered as a failure (0). The three‐month hip function score provides a convenient measure of the success or failure of the procedure in our hypothetical phase II trial.

For K=2, the bivariate density function in Eq. (5) is $h_{p}\left(p_{1},p_{2}\right)=f_{p}\left(p_{1}\right)f_{p}\left(p_{2}\right)\left(1+\omega \phi \left(p_{1}\right)\phi \left(p_{2}\right)\right)$ where $\phi (p_{k})$ is taken to be $p_{k}-\mu_{k},k=1,2$, and $\mu_{k}$ is the expected value of $p_{k}$. The correlation coefficient of *p*_1_ and *p*_2_ is given by $\rho =\omega \sigma_{1}\sigma_{2}$ where $\sigma_{k}^{2}$ is the variance of $p_{k}$ and the mixing parameter, ω, satisfies the condition

(7) $$\max\left\{\frac{-1}{\mu_{1}\mu_{2}},\frac{-1}{\left(1-\mu_{1}\right)\left(1-\mu_{2}\right)}\right\}\leq \omega \leq \min\left\{\frac{1}{\mu_{1}\left(1-\mu_{2}\right)},\frac{1}{\mu_{2}\left(1-\mu_{1}\right)}\right\},$$

so that ρ∈[−1,1] (Lee, 1996). A range of prior distributions of this form are considered below.

### 6.3 Illustration of the bivariate case

From the published results (Costa et al., 2012), we assume for designing the series of trials that the probability of success of the control treatment is $p_{C}=0.5$. We also assume that the probability of each treatment follows the same marginal prior distribution, as we believe a priori that both treatments are equally effective. In order to understand the operating characteristics of the design we consider the following prior density functions; Beta(1,1) which is equivalent to obtaining information from a uniform distribution, Beta(0.12,0.08),Beta(3,2),Beta(12,8), and Beta(143.4,95.6) which have expected value 0.6, indicating a prior belief that the experimental treatment efficacy is greater than $p_{C}$, but variances ranging from 0.20 to 0.001. We also consider prior distributions with expected value less than $p_{C}$, namely, Beta(8,12) and Beta(8.792,11.65) which have the same variance of 0.0114 but different expected values, 0.40 and 0.43, respectively, and Beta(2,3) and Beta(7,10.5) which have the same expected value, 0.40, but different variances, 0.04 and 0.0130, respectively. Note that it is unlikely in practice to select a highly informative prior such as Beta(12,8) and Beta(143.4,95.6) or a U‐shaped distribution such as Beta(0.12,0.08) and Beta(0.84,0.56) but they are included here for illustration.

We also need to make some (albeit fairly crude) assumptions about the costs of different aspects of the design. For simplicity, the fixed and variable costs for both treatments are assumed to be equal for the two experimental treatments. Assume that a reward when the phase III trial is successful (new treatment is statistically significant better than the control treatment at two sided α=0.05 level) is a gain of *U* = £3 million, and costs of setting up and the personnel needed for a phase II and III trials are l II ,k = £30 000 and l III ,k = £300 000, respectively. The cost per patient in both phase II and III setting is set to be equal, that is, c II ,k=c III ,k = £750.

We computed the optimal designs and strategies with the following values: a projected total size of the population, $N=350,m_{ki}=5$, for all *k* and *i* (the subscripts are suppressed from henceforth), and the minimum size of phase III, n III ,min=300 for prior distributions stated above and different values of ω=0and4 where the latter value is the maximum integer that satisfies the condition shown in (7) and the former is when the treatment efficacy is independent.

**Figure 1:**
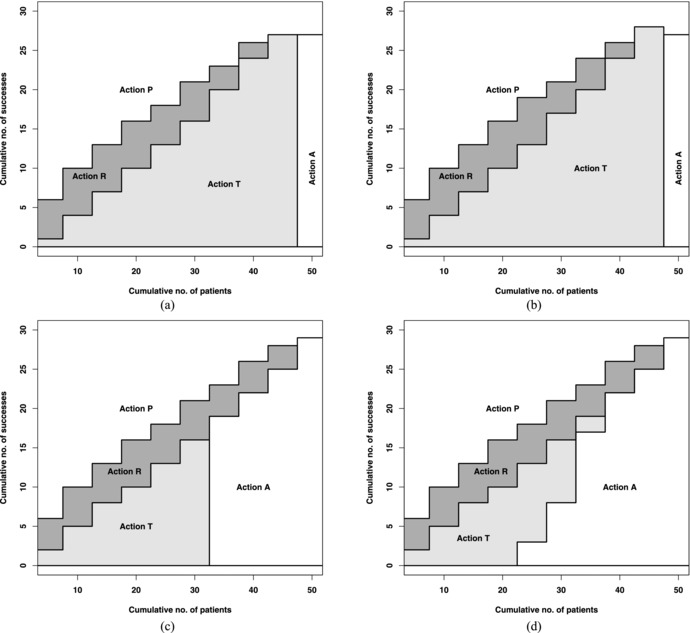
Decision rules for optimal actions for the first phase II trial based on (a) Beta(1,1) and ρ=0 (equivalently, ω=0), (b) Beta(1,1) and ρ=0.33 (equivalently, ω=4), (c) Beta(8.792,11.65) and ρ=0 (equivalently, ω=0), and (d) Beta(8.792,11.65) and ρ=0.046 (equivalently, ω=4)

Figure 1 shows the decision scheme for optimal actions for the first phase II trial for Beta(1,1) and Beta(8.792,11.65). As an illustration, Fig. 1(b) shows that if there were at least one success out of the first five patients then the optimal action is action R (continue recruitment to the current trial). If no success is observed then the optimal action is action T (to stop the current trial and start a new phase II trial with the second treatment). The minimum number of patients needed to proceed to the phase III trial (action P) is 10 (of which all must be successes) and the maximum number needed is 45 (of which at least 28 must be successes).

Table 1 shows the operating characteristics of the development plan for various prior densities. We sampled data from a Bernoulli distribution with p=0.52, the proportion of patients from WAT resurfacing arthroplasty (experimental arm) with good/excellent function at three months postsurgery, 1000 times for each scenario. For each simulation, batches (size m=5) were generated in turn and the optimum action, determined from the decision scheme, taken, for each of the 10 scenarios (R code available as a web supplement). During the first trial, after accumulating data from each batch, the available actions were to continue to accumulate data for treatment 1, to take action T, to take action P, or to stop the development program (action A). During the trial for treatment 2, action T was not available. The number of occasions that various terminal actions were taken for the first and second treatments, the median and range of the number of participants needed are also summarized. The same simulated data were used for each scenario, allowing us to make direct comparison between scenarios.

**Table 1:** Number of optimal terminal actions taken after sampling 1000 times from a Bernoulli distribution with p=0.52 for various prior distributions, Beta(a,b), and mixing parameter, ω (equivalently, correlation, ρ). Available terminal actions based on accumulated data at treatment 1 were to start a new phase II study (action T), move to a phase III study (action P), or abandon the development program (action A); only the latter two options were available at treatment 2. The proportion of times action P or A was taken at treatment 2 is in brackets. The median and range of the number of study participants needed to make a terminal action are shown for both treatments

			Treatment 1					Treatment 2				
			Action					Action				Expected gain
(a,b)	ω	ρ	T	P	A	Median	Range	P	A	Median	Range	G Total (s10,n10,N)
(0.12, 0.08)	0	0	951	49	0	25	(5, 45)	577 (0.61)	374 (0.39)	15	(5, 45)	0.6478
	4	0.800	968	32	0	25	(5, 45)	533 (0.55)	435 (0.45)	20	(5, 45)	0.5007
(0.84, 0.56)	0	0	943	57	0	25	(5, 45)	548 (0.58)	395 (0.42)	20	(5, 45)	0.5839
	4	0.400	943	57	0	25	(5, 45)	534 (0.57)	409 (0.43)	20	(5, 45)	0.5224
(3, 2)	0	0	907	93	0	25	(5, 45)	584 (0.64)	323 (0.36)	20	(5, 45)	0.5139
	4	0.160	907	93	0	25	(5, 45)	571 (0.63)	336 (0.37)	20	(5, 45)	0.4927
(12, 8)	0	0	880	120	0	25	(5, 45)	693 (0.79)	187 (0.21)	15	(5, 45)	0.4172
	4	0.046	874	126	0	25	(5, 45)	684 (0.78)	190 (0.22)	10	(5, 45)	0.4123
(143.4, 95.6)	0	0	621	379	0	10	(5, 45)	621 (1.00)	0 (0.00)	5	(5, 5)	0.2832
	4	0.004	621	379	0	10	(5, 45)	621 (1.00)	0 (0.00)	5	(5, 5)	0.2832
(1, 1)	0	0	858	142	0	25	(5, 45)	479 (0.56)	379 (0.44)	15	(5, 45)	0.4395
	4	0.333	899	101	0	25	(5, 45)	521 (0.58)	378 (0.42)	20	(5, 45)	0.3955
(8.792, 11.65)	0	0	647	173	180	20	(5, 50)	115 (0.18)	532 (0.82)	20	(5, 45)	0.0130
	4	0.046	696	173	131	20	(5, 50)	112 (0.16)	584 (0.84)	20	(5, 45)	0.0125
(2, 3)	0	0	781	219	0	25	(5, 45)	357 (0.46)	424 (0.54)	20	(5, 45)	0.1705
	4	0.160	803	197	0	25	(5, 45)	353 (0.44)	450 (0.56)	20	(5, 45)	0.1614
(7, 10.5)	0	0	469	132	399	25	(5, 50)	77 (0.16)	392 (0.84)	25	(5, 45)	0.0041
	4	0.052	466	132	402	25	(5, 50)	74 (0.16)	392 (0.84)	25	(5, 45)	0.0036
(8, 12)	0	0	–	–		–	–	–	–	–	–	−0.0022
	4	0.046	–	–	–	–	–	–	–	–	–	−0.0022

The estimated proportion of success from the WAT resurfacing arthroplasty arm was 0.52; slightly higher than the historical control $p_{C}=0.50$ we assumed for the trial design but lower than 0.60, the expected value of some of the informative prior densities shown in Table 1. The proportion of times action P was taken increased as the variance of the prior density decreases (from 0.20 to 0.001) because the minimum threshold to take action P lowers accordingly. In the case of prior Beta(8,12) whose expected value (0.40) was less than $p_{C}$ the expected gain was −0.0022, that is, it was not worthwhile starting the program at all.

As we see from Fig. 1, the optimal decision schemes for treatment 1 do not differ much when both treatments have the same positive prior and the correlation changes from zero to nonzero, and we observed similar operating characteristics in Table 1 where the proportions of actions T and P taken at treatment 1 were identical or very similar regardless of the correlation. The correlation affects the expected utility of the second treatment because responses from treatment 1 inform the prior of treatment 2. In Fig. 1(c), the treatment effects are independent and so the expected utility of action T which depends on the number of patients recruited to the first treatment is constant because it does not depend on the responses from treatment 1. Thus, there is a straight cut‐off between taking actions T and A. However, when there is some correlation between the treatment effects (see, Fig. 1(d)), the expected utility of action T depends on the responses from the first treatment and so we observe a more gradual shift in its value as the number of successes changes. Supporting Information Figs. S1–S3 show the decision schemes for treatment 2 after observing responses from $n_{1+}=10$ patients from treatment 1 for marginal priors Beta(3,2),Beta(1,1), and Beta(2,3) when the treatment effects are independent and correlated. The continuation region for treatment 2 changes subtly depending on the number of successes, $s_{1+}$, when the treatment effects are correlated.

### 6.4 Trivariate case

We also explore the feasibility and characteristics of the design when K=3. Lee (1996) showed that any subvector $\left(p_{k_{1}^{{}^{\prime }}},\ldots ,p_{k_{m}^{{}^{\prime }}}\right),1\leq k_{1}^{{}^{\prime }}<k_{2}^{{}^{\prime }}<\ldots <k_{m}^{{}^{\prime }}\leq K$ has a joint density of the form (5). This implies that $1+R_{\Omega_{k_{m}^{{}^{\prime }}}}\left(p_{k_{1}^{{}^{\prime }}},\ldots ,p_{k_{m}^{{}^{\prime }}}\right)\geq 0$. Therefore, we assume the pairwise mixing parameters, $\omega_{12},\omega_{13}$, and ω_23_ are bounded as in (7) and the lower and upper bounds of ω_123_ are given in Supporting Information Appendix A.

Following earlier illustrations, we consider all treatments' efficacies to have the same marginal beta prior density, $p_{k}∼Beta\left(a_{k},b_{k}\right),k=1,2,3$, with $a_{k}=a$ and $b_{k}=b$ and $\omega_{12}=\omega_{13}=\omega_{23}=\omega$. As shown in the Supporting Information Appendix A, the lower and upper bounds for ω_123_ are

(8) $$\begin{array}{ccc} & & \max\left\{\frac{-[1+3\omega {\left(1-\mu \right)}^{2}]}{{\left(1-\mu \right)}^{3}},\frac{-[1+\omega \mu^{2}-2\omega \mu \left(1-\mu \right)]}{\mu^{2}\left(1-\mu \right)}\right\}\\ & & \;\leq \omega_{123}\leq \min\left\{\frac{1+3\omega \mu^{2}}{\mu^{3}},\frac{1-2\omega \mu \left(1-\mu \right)+\omega {\left(1-\mu \right)}^{2}}{\mu {\left(1-\mu \right)}^{2}}\right\}. \end{array}$$

**Figure 2:**
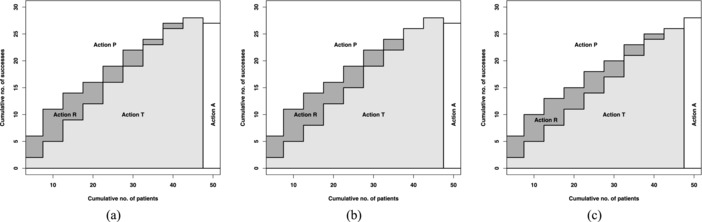
Decision rules for optimal actions for the first phase II trial based on (a) $Beta\left(3,2\right),\omega_{12}=\omega_{13}=\omega_{23}=4$ and $\omega_{123}=-3$, (b) $Beta\left(1,1\right),\omega_{12}=\omega_{13}=\omega_{23}=4$ and $\omega_{123}=0$, and (c) $Beta\left(2,3\right),\omega_{12}=\omega_{13}=\omega_{23}=4$ and $\omega_{123}=3$ when there are K=3 treatments available

Figure 2 shows the decision scheme for the first treatment for a=3,b=2 with ω=4 and $\omega_{123}=-3$ (Fig. 2a), a=1,b=1 with ω=4 and $\omega_{123}=0$ (Fig. 2b), and a=2,b=3 with ω=4 and $\omega_{123}=3$ (Fig. 2c). Analogous to the bivariate case, the pairwise mixing parameter, ω, takes the maximum integer that satisfies condition (7) and the trivariate mixing parameter, ω_123_ takes the maximum integer that satisfies condition (8).

In the trivariate case, the continuation region (action C) shifts up suggesting that more successes are needed before stopping the trial and proceeding to a phase III trial with the first treatment. Otherwise, the optimal action is to try subsequent treatments. The expected utilities of the whole program, G Total (s10,n10,N) are 0.555, 0.454, and 0.203 for Beta(3,2),Beta(1,1), and Beta(2,3) prior distributions, respectively. They are higher than the bivariate case which is expected as in this setting we have more treatments to learn from.

## 7 Discussion

### 7.1 Correlation and prior distributions

The predecessor of this design, proposed by Hee and Stallard (2012), does not consider the correlation between the efficacy of the treatments. We showed here that because of the correlation, responses from the first treatment update the prior density of the second treatment and consequently, the optimal decision scheme for the second treatment is more sensitive to an ineffective treatment, that is, the action C region shifts higher when there is correlation, making it more difficult to proceed to a phase III trial with the second treatment but easier to stop the second trial and abandon the whole program than when it is independent (see, Supporting Information Figs. S1–S3).

The Sarmanov family of distributions is slightly more flexible than those of the Farlie–Gumbel–Morgenstern (FGM) distributions; Lee (1996) showed that the correlation coefficients of the bivariate Sarmanov have wider ranges. However, when both marginal prior distributions are identical beta distributions with parameters a,b≥1, the correlation is limited to the interval [−1/3,1/3], the same limitation as the FGM distributions. As the limit of the level of correlation is low there is little difference in the operating characteristics between correlated and independent cases as seen in the expected gain of the whole development program (Table 1). The bivariate beta distribution proposed by Olkin and Trikalinos (2015) is more flexible than the Sarmanov form as it allows correlations over the full range [‐1,1] has no closed form expression. Therefore, the posterior distribution could only be calculated numerically. The number of terms in its joint density increases by order of $2^{k}-1$ which may be more computationally inconvenient than Sarmanov when K>3.

In our illustrations the prior densities for both treatments are assumed to be the same. Nevertheless, the order in which the treatments are entered into the study is important. For instance, the results from three prior densities that are more reasonable in practice, that is, Beta(3,2),Beta(1,1), and Beta(2,3) indicate that the second treatment is more likely than the first treatment to proceed to a phase III setting. This suggests that as the number of patients available for phase II becomes smaller there is a higher probability of proceeding to a phase III trial than would have been the case, all other things being equal, earlier in the program.

In some settings, such as a scenario where a large funding body is funding a series of clinical trials with treatments from different drug classes for the same population, the prior densities may be different. It can be seen that again the ordering of treatments to be tested in the program is important with similar characteristics as when both treatments have the same marginal prior density, that is, the second treatment has a higher chance to proceed to a phase III setting (see Supporting Information Table S1). Supporting Information Table S1 presents results from simulations under four scenarios; two of which started with a treatment with a more informative prior followed by one with a less informative prior and the other two scenarios vice versa. Based on the expected utility of the whole program, treatments with less informative prior densities should be selected as the first treatments for the program. We achieve higher expected utility because we learn more about treatment efficacy.

The challenge in a Bayesian methodology is the specification of the prior distribution which should reflect one's belief in the values of parameters of the prior distribution of the treatment effect. In the specification of the prior distribution one may estimate the mean of the treatment efficacy and its variance from previously conducted studies as suggested above. In the absence of data, one may elicit the prior distributions and there is a considerable literature on this area (see, e.g., Chaloner, Church, Louis, & Matts, 1993; O'Hagan, 1998) and case studies by Hampson, Whitehead, Eleftheriou, and Brogan (2014); Kinnersley and Day (2013). In our illustration, the mixing parameters, $\omega_{ij}$, and equivalently, the correlation coefficients, ρ, were chosen in order to compare highly correlated treatment effects with independent cases. We envisage that the elicitation of the correlation coefficient to be conducted at the same time as the prior distributions. A possibility is to elicit from experts whether or not their beliefs of the density of *p*_2_ would change given the marginal of *p*_1_ and responses from $n_{1+}$ patients and the magnitude and direction of any change.

### 7.2 Expected utility

The utility functions we proposed here may be modified to represent similarly realistic alternatives in other settings. For example, when action A taken the program is abandoned and patients yet to be recruited to the trial would continue with their standard treatment. The utility of action A could include the gain that the remaining $N-\sum_{j=1}^{k-1}n_{j+}-n_{ki}$ patients could get from the standard treatment less the cost of patients recruited to the *k*‐th trial so far, that is, GA(k,p,nki,N)=Us(N−∑j=1k−1nj+−nki)−c II ,knki, where $U_{s}$ is the gain achieved from the standard treatment and may be set to a fixed value that is relative to *U* or a function of the efficacy of the standard treatment. In principle, $U_{s}$ could be a function of the treatment efficacy and then the expected gain of action A is obtained by taking expectation over all possible values of the true efficacy of the standard treatment. We could also consider the loss incurred from taking a wrong decision, for example, abandoning the whole program (action A) when one of the experimental treatments is actually better than the standard treatment by making $U_{s}$ a function of all parameters. The utility function can also be defined as the benefit of health outcome such as quality of life, for example, expressed in quality‐adjusted life years (QALY).

### 7.3 Computational time

It is undoubtedly more challenging to run a series of decision‐theoretic trials such as we proposed than a fixed design as recruitment may need to be suspended while patients' responses are collected before a decision is made. However, the time taken to stop the current treatment and try a new one is reduced if the decision‐theoretic trial stops early. An additional challenge is that the computation is complex and time consuming with dynamic programming. This complexity is compounded further by the correlation between the unknown parameters, p. The calculation of expected utilities of each action at each stage depends on the posterior density of p which depends on responses from all preceding trials and stages within them. The computation time increases rapidly with increasing number of trials and number of interim stages; the computation time is five times longer when the number of stages increases from 10 to 20, and 42 times longer from 10 to 30. The expected utility of the program, as expected, increases as *m* decreases. In a bivariate example with prior Beta(3,2) keeping all values to be same as above except N=360 and for different values of m=5,10,15,20, that is, 12 to 3 interim stages, the expected utilities are 0.4963, 0.4950, 0.4932, and 0.4913 (results not shown). In our trivariate illustration, the computational time increases from a few minutes to a few hours.

### 7.4 Limitation

A limitation of our proposed design is that the primary endpoints for phase II and III trials are binary. This may not be true for trials in other disease areas such as oncology where it is more common to have binary response in phase II but time‐to‐event outcome in phase III. Similarly, other rare diseases may have continuous or time‐to‐event outcome as the primary endpoint in the phase III trial. In these cases, the Sarmanov beta‐binomial distribution is still applicable in modeling the correlation between treatments but an additional joint model is necessary to indicate the association between the phase II endpoint and the phase III endpoint. This is the topic of ongoing work. Another scenario is when the endpoints in phases II and III are the same but are not binary. In such case, an alternative to the Sarmanov distribution would be necessary to model the correlation between endpoints for different treatments.

### 7.5 Further work

Although not limited to this case, as shown by the illustration of a surgical trial, the sequential design proposed in this paper is motivated by the scenario of a small population such as a rare disease where although there is more than one treatment available for a clinical trial only one can be evaluated at a time because of the limited resources. In a larger population, there is an option to run experimental treatments concurrently. Designs proposed by, for example, Bretz, Schmidli, König, Racine, and Maurer (2006); Hobbs, Chen, and Lee (2016); Rossell et al. (2007); Stallard and Thall (2001); and Stallard and Todd (2003), allow a few treatments to be tested concurrently with treatment dropped at interim stages for futility. In particular, designs by Rossell et al. (2007), and Stallard and Thall (2001) are based on optimal decision‐theoretic framework. The Sarmanov multivariate distribution is capable in accommodating concurrently run trials as the posterior joint distribution is obtained as shown in (6) but the backward induction will be computationally complex.

An alternative to the proposed design is to assume that $p_{1},\ldots ,p_{K}$ are conditionally independently and identically distributed with Beta(a,b) and we assume an appropriate hyperprior distribution for the parameters (a,b). The interest is then to find the joint posterior density of the hyperparameters (a,b) which then estimates the density function of $p_{k}$. Designs proposed by Ding et al. (2008) and Rossell et al. (2007) make use of this hierarchical model where one does not need to explicitly define the correlation between probability of success to update the density function of another parameter.

One of the assumptions underlying this development plan is that treatments that have been tested and subsequently abandoned cannot be evaluated further in the same program. A possible extension to this program is to allow the old treatments to rejoin the program in the phase III evaluation. In this setting, one may have additional actions to choose in each interim stage, that is, an action where the current phase II trial is stopped and a phase III trial is initiated with the rejoined treatment.

## Conflict of interest

The authors have declared no conflict of interest.

## Supporting information

Supporting Information bimj1791‐sup‐0001‐CorrelatedTreatmentEffects_RCode_Hee_et_al.zip

Supporting Information bimj1791‐sup‐0002‐SuppMat.pdf
