# Supplementary material for: Decision‐theoretic designs for a series of trials with correlated treatment effects using the Sarmanov multivariate beta‐binomial distribution
Source: Biom J. 2017 Jul 26;60(2):232–45. doi: 10.1002/bimj.201600202 (PMC5888217; doi:10.1002/bimj.201600202)
Supplement: Supplementary file 2 — Supporting Information bimj1791‐sup‐0002‐SuppMat.pdf [file BIMJ-60-232-s002.pdf]

Web-based supplementary materials for

Decision-theoretic designs for a series of trials with correlated treatment effects using the Sarmanov multivariate beta-binomial distribution

by

Siew Wan Hee, Nicholas Parsons and Nigel Stallard

Web Appendix A: Multivariate Sarmanov distribution

Let $f(p_k), k = (1, \ldots, K)$ be the univariate probability density function and $h(p_1, \ldots, p_K)$ is the multivariate joint density function. Lee (1996) showed that the Sarmanov multivariate joint density has the form

$$h(p_1, \ldots, p_K) = \prod_{k=1}^{K} f(p_k)\big(1 + R_{\Omega_K}(p_1, \ldots, p_K)\big) \tag{1}$$

with

$$\begin{aligned} R_{\Omega_K}(p_1, \ldots, p_K) = &\sum_{j_1=1}^{K-1} \sum_{j_2=j_1+1}^{K} \omega_{j_1,j_2} \phi(p_{j_1})\phi(p_{j_2}) \\ &+ \sum_{j_1=1}^{K-2} \sum_{j_2=j_1+1}^{K-1} \sum_{j_3=j_2+1}^{K} \omega_{j_1,j_2,j_3} \phi(p_{j_1})\phi(p_{j_2})\phi(p_{j_3}) \\ &+ \cdots + \omega_{1,2,\ldots,K} \prod_{j=1}^{K} \phi(p_j), \end{aligned}$$

where $\Omega_K = \{\omega_{j_1,j_2}, \omega_{j_1,j_2,j_3}, \ldots, \omega_{1,2,\ldots,K}\}$ is the mixing parameter and following Lee's definition, $\phi(p_k)$ is a nonconstant mixing function bounded by $\int_{-\infty}^{\infty} \phi(p_k) f_p(p_k)\, dp_k = 0$. The set of real numbers Ω_K is chosen such that

$$1 + R_{\Omega_K}(p_1, \ldots, p_K) \geq 0 \tag{2}$$

holds for all $p_i \in \mathbb{R}, i = 1, \ldots, K$.

Lee also showed that any subvector $(p_{k_1'}, \ldots, p_{k_m'}), 1 \leq k_1' < k_2' < \ldots < k_m' \leq K$ has a joint density of the form as in (1). This implies that elements of Ω_K that satisfy the condition in (2) should also satisfy $1 + R_{\Omega_{k_m'}}(p_{k_1'}, \ldots, p_{k_m'}) \geq 0$.

Consider a trivariate case, let the mixing function $\phi(p_k) = p_k - \mu_k, k = 1, 2, 3$ where $p_k \in [0, 1]$ and μ_k is the expected value of p_k then

$$\begin{aligned} &1 + \omega_{12}(p_1 - \mu_1)(p_2 - \mu_2) + \omega_{13}(p_1 - \mu_1)(p_3 - \mu_3) \\ &\quad + \omega_{23}(p_2 - \mu_2)(p_3 - \mu_3) + \omega_{123}(p_1 - \mu_1)(p_2 - \mu_2)(p_3 - \mu_3) \geq 0. \end{aligned} \tag{3}$$

To find the lower and upper bounds of each element of Ω_3 such that the condition is satisfied, we could substitute (p_1, p_2, p_3) with extreme points $(0, 0, 0), (0, 0, 1), (0, 1, 0), (0, 1, 1), (1, 0, 0), (1, 0, 1), (1, 1, 0)$ and $(1, 1, 1)$ to equation (3):

$$(0,0,0): \quad \omega_{123} \leq \frac{1 + \omega_{12}\mu_1\mu_2 + \omega_{13}\mu_1\mu_3 + \omega_{23}\mu_2\mu_3}{\mu_1\mu_2\mu_3} \tag{4}$$

$$(0,1,1): \quad \omega_{123} \leq \frac{1 - \omega_{12}\mu_1(1-\mu_2) - \omega_{13}\mu_1(1-\mu_3) + \omega_{23}(1-\mu_2)(1-\mu_3)}{\mu_1(1-\mu_2)(1-\mu_3)} \tag{5}$$

$$(1,0,1): \quad \omega_{123} \leq \frac{1 - \omega_{12}(1-\mu_1)\mu_2 + \omega_{13}(1-\mu_1)(1-\mu_3) - \omega_{23}\mu_2(1-\mu_3)}{(1-\mu_1)\mu_2(1-\mu_3)} \tag{6}$$

$$(1,1,0): \quad \omega_{123} \leq \frac{1 + \omega_{12}(1-\mu_1)(1-\mu_2) - \omega_{13}(1-\mu_1)\mu_3 - \omega_{23}(1-\mu_2)\mu_3}{(1-\mu_1)(1-\mu_2)\mu_3} \tag{7}$$

$$(0,0,1): \quad \omega_{123} \geq -\frac{1 + \omega_{12}\mu_1\mu_2 - \omega_{13}\mu_1(1-\mu_3) - \omega_{23}\mu_2(1-\mu_3)}{\mu_1\mu_2(1-\mu_3)} \tag{8}$$

$$(0,1,0): \quad \omega_{123} \geq -\frac{1 - \omega_{12}\mu_1(1-\mu_2) + \omega_{13}\mu_1\mu_3 - \omega_{23}(1-\mu_2)\mu_3}{\mu_1(1-\mu_2)\mu_3} \tag{9}$$

$$(1,0,0): \quad \omega_{123} \geq -\frac{1 - \omega_{12}(1-\mu_1)\mu_2 - \omega_{13}(1-\mu_1)\mu_3 + \omega_{23}\mu_2\mu_3}{(1-\mu_1)\mu_2\mu_3} \tag{10}$$

$$(1,1,1): \quad \omega_{123} \geq -\frac{1 + \omega_{12}(1-\mu_1)(1-\mu_2) + \omega_{13}(1-\mu_1)(1-\mu_3) + \omega_{23}(1-\mu_2)(1-\mu_3)}{(1-\mu_1)(1-\mu_2)(1-\mu_3)} \tag{11}$$

From equations (4) and (8)

$$\begin{aligned} &-\frac{1 + \omega_{12}\mu_1\mu_2 - \omega_{13}\mu_1(1-\mu_3) - \omega_{23}\mu_2(1-\mu_3)}{\mu_1\mu_2(1-\mu_3)} \\ &\qquad\qquad \leq \frac{1 + \omega_{12}\mu_1\mu_2 + \omega_{13}\mu_1\mu_3 + \omega_{23}\mu_2\mu_3}{\mu_1\mu_2\mu_3} \\ &-\mu_3(1 + \omega_{12}\mu_1\mu_2) + \mu_3(1-\mu_3)(\omega_{13}\mu_1 + \omega_{23}\mu_2) \\ &\qquad\qquad \leq (1-\mu_3)(1 + \omega_{12}\mu_1\mu_2) + \mu_3(1-\mu_3)(\omega_{13}\mu_1 + \omega_{23}\mu_2) \\ &\qquad\quad 0 \leq 1 + \omega_{12}\mu_1\mu_2 \\ &\quad \frac{-1}{\mu_1\mu_2} \leq \omega_{12} \end{aligned}$$

Similarly, from the following equations, the lower and upper bounds for the pairwise

mixing parameters are

$$
\begin{aligned}
(7) \text{ and } (11): &\quad \frac{-1}{(1-\mu_1)(1-\mu_2)} &\le \omega_{12} \\
(4) \text{ and } (9): &\quad \frac{-1}{\mu_1\mu_3} &\le \omega_{13} \\
(6) \text{ and } (11): &\quad \frac{-1}{(1-\mu_1)(1-\mu_3)} &\le \omega_{13} \\
(4) \text{ and } (10): &\quad \frac{-1}{\mu_2\mu_3} &\le \omega_{23} \\
(5) \text{ and } (11): &\quad \frac{-1}{(1-\mu_2)(1-\mu_3)} &\le \omega_{23} \\
(5) \text{ and } (9): &\quad \omega_{12} &\le \frac{1}{\mu_1(1-\mu_2)} \\
(6) \text{ and } (10): &\quad \omega_{12} &\le \frac{1}{(1-\mu_1)\mu_2} \\
(5) \text{ and } (8): &\quad \omega_{13} &\le \frac{1}{\mu_1(1-\mu_3)} \\
(7) \text{ and } (10): &\quad \omega_{13} &\le \frac{1}{(1-\mu_1)\mu_3} \\
(6) \text{ and } (8): &\quad \omega_{23} &\le \frac{1}{\mu_2(1-\mu_3)} \\
(7) \text{ and } (9): &\quad \omega_{23} &\le \frac{1}{(1-\mu_2)\mu_3}.
\end{aligned}
$$

Henceforth, the lower and upper bounds for $\omega_{ij}, i, j = 1, 2, 3$ and $i \neq j$,

$$
\max\left\{\frac{-1}{\mu_i\mu_j}, \frac{-1}{(1-\mu_i)(1-\mu_j)}\right\} \le \omega_{ij} \le \min\left\{\frac{1}{\mu_i(1-\mu_j)}, \frac{1}{(1-\mu_i)\mu_j}\right\}
$$

which is the case in the bivariate Sarmanov density.

From equations (4) to (11) the lower and upper bounds for ω_{123} are

$$\begin{aligned}
\max\Big\{ & -\frac{1+\omega_{12}\mu_1\mu_2-\omega_{13}\mu_1(1-\mu_3)-\omega_{23}\mu_2(1-\mu_3)}{\mu_1\mu_2(1-\mu_3)}, \\
& -\frac{1-\omega_{12}\mu_1(1-\mu_2)+\omega_{13}\mu_1\mu_3-\omega_{23}(1-\mu_2)\mu_3}{\mu_1(1-\mu_2)\mu_3}, \\
& -\frac{1-\omega_{12}(1-\mu_1)\mu_2-\omega_{13}(1-\mu_1)\mu_3+\omega_{23}\mu_2\mu_3}{(1-\mu_1)\mu_2\mu_3}, \\
& -\frac{1+\omega_{12}(1-\mu_1)(1-\mu_2)+\omega_{13}(1-\mu_1)(1-\mu_3)+\omega_{23}(1-\mu_2)(1-\mu_3)}{(1-\mu_1)(1-\mu_2)(1-\mu_3)}\Big\} \\
& \leq \omega_{123} \leq \min\Big\{\frac{1+\omega_{12}\mu_1\mu_2+\omega_{13}\mu_1\mu_3+\omega_{23}\mu_2\mu_3}{\mu_1\mu_2\mu_3}, \\
& \qquad \frac{1-\omega_{12}\mu_1(1-\mu_2)-\omega_{13}\mu_1(1-\mu_3)+\omega_{23}(1-\mu_2)(1-\mu_3)}{\mu_1(1-\mu_2)(1-\mu_3)}, \\
& \qquad \frac{1-\omega_{12}(1-\mu_1)\mu_2+\omega_{13}(1-\mu_1)(1-\mu_3)-\omega_{23}\mu_2(1-\mu_3)}{(1-\mu_1)\mu_2(1-\mu_3)}, \\
& \qquad \frac{1+\omega_{12}(1-\mu_1)(1-\mu_2)-\omega_{13}(1-\mu_1)\mu_3-\omega_{23}(1-\mu_2)\mu_3}{(1-\mu_1)(1-\mu_2)\mu_3}\Big\}.
\end{aligned} \tag{12}$$

When $a_1 = a_2 = a_3 = a$ and $b_1 = b_2 = b_3 = b$ then $\mu_1 = \mu_2 = \mu_3 = \mu = a/(a+b)$ and $\omega_{12} = \omega_{13} = \omega_{23} = \omega$. Equation (12) becomes

$$\begin{aligned}
\max\Big\{\frac{-[1+3\omega(1-\mu)^2]}{(1-\mu)^3}, \frac{-[1+\omega\mu^2-2\omega\mu(1-\mu)]}{\mu^2(1-\mu)}\Big\} \\
\leq \omega_{123} \leq \min\Big\{\frac{1+3\omega\mu^2}{\mu^3}, \frac{1-2\omega\mu(1-\mu)+\omega(1-\mu)^2}{\mu(1-\mu)^2}\Big\}.
\end{aligned}$$

Web Appendix B: Operating Characteristics of the Development Plan

Web Table 1 shows the operating characteristics of the development plan for bivariate Sarmanov beta density when the marginal prior densities of the first and second treatments are not identical.

Web Table 1

Number of optimal terminal actions taken after sampling 1000 times from a Bernoulli distribution with $p = 0.52$ for various prior distributions, $Beta(a, b)$, and mixing parameter, ω (equivalently, correlation, ρ). Available terminal actions based on accumulated data at treatment 1 were to start a new phase II study (action T), move to a phase III study (action P) or abandon the development programme (action A); only the latter two options were available at treatment 2. The proportion of times action P or A was taken at treatment 2 is in brackets. The median and range of the number of study participants needed to make a terminal action are shown for both treatments.

		Treatment 1						Treatment 2					Expected gain
			Action						Action				
ω	ρ	(a, b)	T	P	A	Median	Range	(a, b)	P	A	Median	Range	$\mathcal{G}_{\text{Total}}(\boldsymbol{s_{10}}, \boldsymbol{n_{10}}, N)$
0	0	$(3, 2)$	855	145	0	25	(5, 45)	$(1, 1)$	494 (0.58)	361 (0.42)	15	(5, 45)	0.475
4	0.231	$(3, 2)$	851	149	0	25	(5, 45)	$(1, 1)$	455 (0.53)	396 (0.47)	20	(5, 45)	0.446
0	0	$(1, 1)$	912	88	0	25	(5, 45)	$(3, 2)$	574 (0.63)	338 (0.37)	20	(5, 45)	0.488
4	0.231	$(1, 1)$	946	54	0	20	(5, 45)	$(3, 2)$	580 (0.61)	366 (0.39)	20	(5, 45)	0.457
0	0	$(12, 8)$	909	91	0	20	(5, 45)	$(3, 2)$	568 (0.62)	341 (0.38)	20	(5, 45)	0.445
4	0.086	$(12, 8)$	920	80	0	20	(5, 45)	$(3, 2)$	561 (0.61)	359 (0.39)	20	(5, 45)	0.436
0	0	$(3, 2)$	894	106	0	25	(5, 45)	$(12, 8)$	704 (0.79)	190 (0.21)	10	(5, 45)	0.488
4	0.086	$(3, 2)$	890	110	0	25	(5, 45)	$(12, 8)$	675 (0.76)	215 (0.24)	15	(5, 45)	0.477

Web Appendix C: Decision Rules for the Second Phase II Trial

Web Figures 1–3 show the decision schemes of the second treatment for $Beta(3, 2)$, $Beta(1, 1)$ and $Beta(2, 3)$, respectively, assuming that action T was the optimal action after observing responses from $n_{1+} = 10$ patients from the first trial.

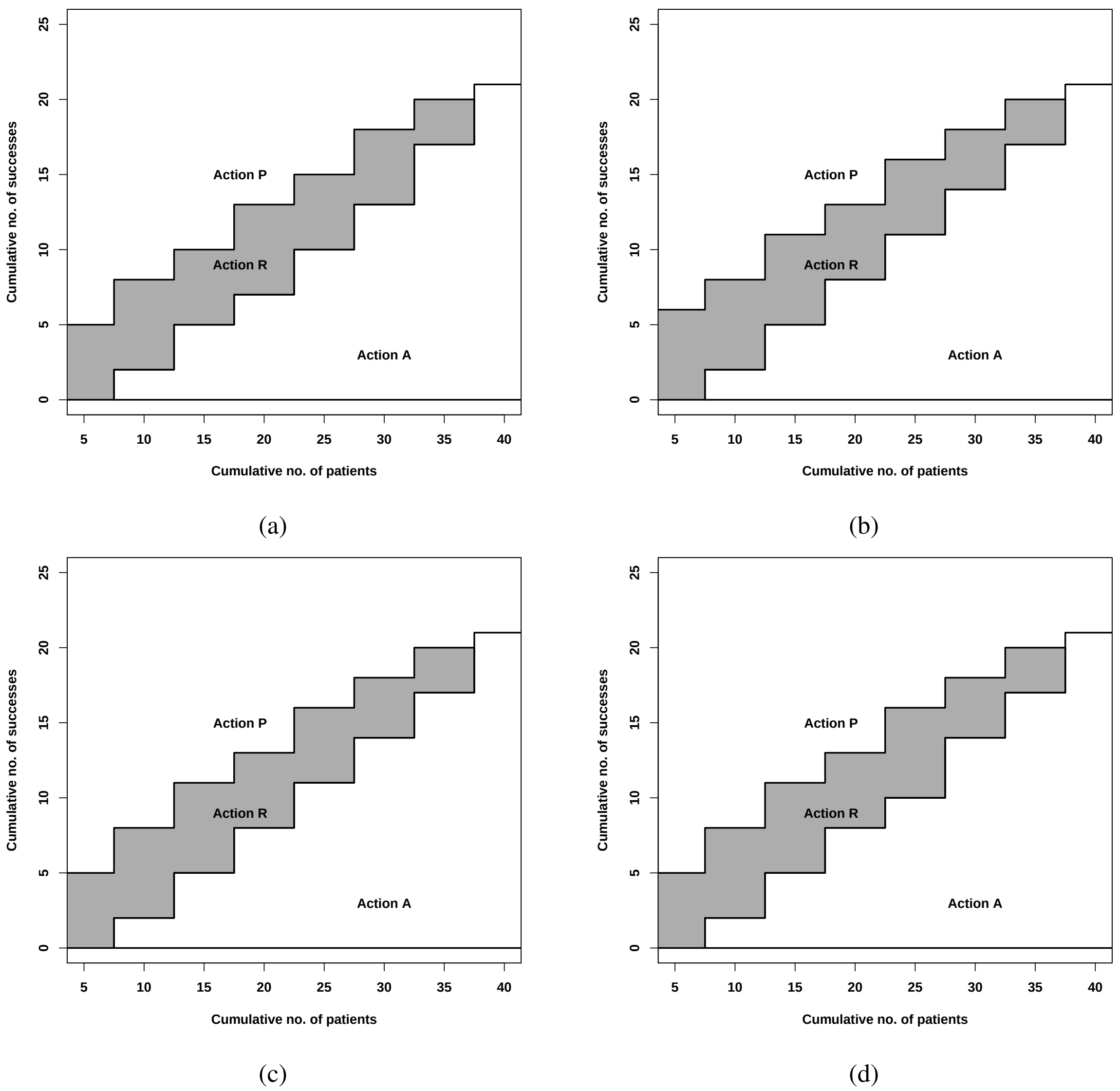

Web Figure 1: Decision rules for optimal actions for the second phase II trial after observing $n_{1+} = 10$ patients from treatment 1 and $p_1, p_2 \sim Beta(3, 2)$ based on (a) independent treatment effects, $\rho = 0$ (equivalently, $\omega = 0$) and (b)–(d) correlated treatment effects, $\rho = 0.16$ (equivalently, $\omega = 4$). In the correlated case, assuming (b) $s_{1+} = 1$, (c) $s_{1+} = 2$ and (d) $s_{1+} = 3$ successes were observed in treatment 1.

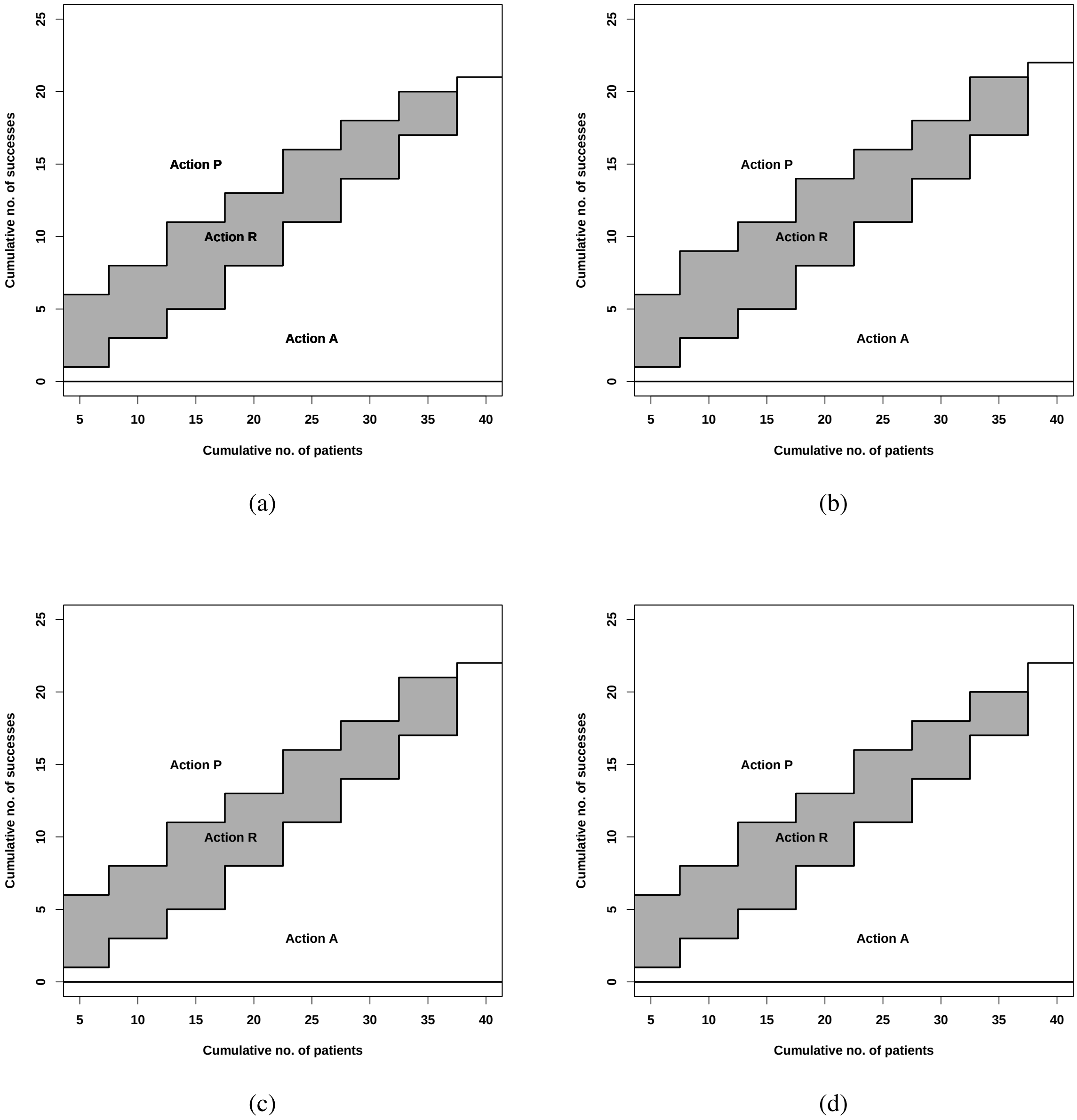

Web Figure 2: Decision rules for optimal actions for the second phase II trial after observing $n_{1+} = 10$ patients from treatment 1 and $p_1, p_2 \sim Beta(1,1)$ based on (a) independent treatment effects, $\rho = 0$ (equivalently, $\omega = 0$) and (b)–(d) correlated treatment effects, $\rho = 0.33$ (equivalently, $\omega = 4$). In the correlated case, assuming (b) $s_{1+} = 1$, (c) $s_{1+} = 2$ and (d) $s_{1+} = 3$ successes were observed in treatment 1.

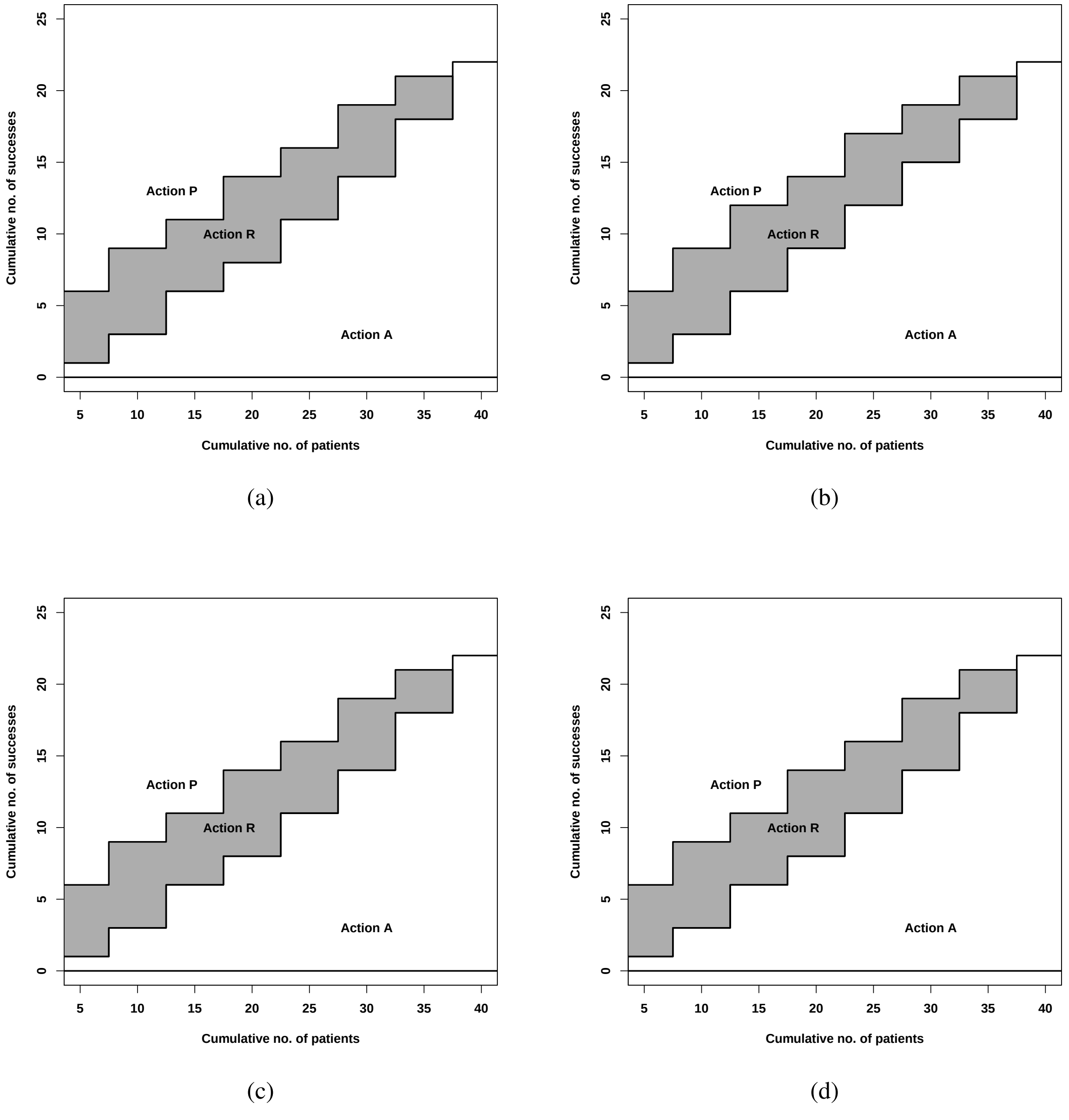

(a) (b) (c) (d)

Web Figure 3: Decision rules for optimal actions for the second phase II trial after observing $n_{1+} = 10$ patients from treatment 1 and $p_1, p_2 \sim Beta(2, 3)$ based on (a) independent treatment effects, $\rho = 0$ (equivalently, $\omega = 0$) and (b)–(d) correlated treatment effects, $\rho = 0.33$ (equivalently, $\omega = 4$). In the correlated case, assuming (b) $s_{1+} = 1$, (c) $s_{1+} = 2$ and (d) $s_{1+} = 3$ successes were observed in treatment 1.
